# Discovering a novel dual specificity tyrosine-phosphorylation-regulated kinase 1A (DYRK1A) inhibitor and its impact on tau phosphorylation and amyloid-β formation

**DOI:** 10.1080/14756366.2024.2418470

**Published:** 2024-11-04

**Authors:** Huang-Ju Tu, Min-Wu Chao, Cheng-Chung Lee, Chao-Shiang Peng, Yi-Wen Wu, Tony Eight Lin, Yu-Wei Chang, Shih-Chung Yen, Kai-Cheng Hsu, Shiow-Lin Pan, Wei-Chun HuangFu

**Affiliations:** a Graduate Institute of Cancer Biology and Drug Discovery, College of Medical Science and Technology, Taipei Medical University, Taipei, Taiwan; b School of Medicine, College of Medicine, National Sun Yat-Sen University, Kaohsiung, Taiwan; c Institute of Biopharmaceutical Sciences, College of Medicine, National Sun Yat-Sen University, Kaohsiung, Taiwan; d The Doctoral Program of Clinical and Experimental Medicine, College of Medicine, National Sun Yat-Sen University, Kaohsiung, Taiwan; e The Ph.D. Program for Translational Medicine, College of Medical Science and Technology, Taipei Medical University, Taipei, Taiwan; f Ph.D. Program for Cancer Molecular Biology and Drug Discovery, College of Medical Science and Technology, Taipei Medical University, Taipei, Taiwan; g Department of Traditional Chinese Medicine, Chang Gung Memorial Hospital, Keelung Medical Center, Keelung, Taiwan; h Warshel Institute for Computational Biology, The Chinese University of Hong Kong (Shenzhen), Shenzhen, Guangdong, People’s Republic of China; i TMU Research Center of Cancer Translational Medicine, Taipei Medical University, Taipei, Taiwan

**Keywords:** DYRK1A, structure-based virtual screening, Tau, Aβ

## Abstract

Dual-specificity tyrosine-regulated kinase 1 A (DYRK1A) is crucial in neurogenesis, synaptogenesis, and neuronal functions. Its dysregulation is linked to neurodegenerative disorders like Down syndrome and Alzheimer’s disease. Although the development of DYRK1A inhibitors has significantly advanced in recent years, the selectivity of these drugs remains a critical challenge, potentially impeding further progress. In this study, we utilised structure-based virtual screening (SBVS) from NCI library to discover novel DYRK1A inhibitors. The top-ranked compounds were then validated through enzymatic assays to assess their efficacy towards DYRK1A. Among them, NSC361563 emerged as a potent and selective DYRK1A inhibitor. It was shown to decrease tau phosphorylation at multiple sites, thereby enhancing tubulin stability. Moreover, NSC361563 diminished the formation of amyloid β and offered neuroprotective benefits against amyloid β-induced toxicity. Our research highlights the critical role of selective DYRK1A inhibitors in treating neurodegenerative diseases and presents a promising starting point for the development of targeted therapies.

## Introduction

Dual-specific tyrosine phosphorylation-regulated kinases (DYRKs) play pivotal roles in diverse cellular functions, including cell cycle regulation, mRNA splicing, DNA damage response, and chromatin modulation^1^. The DYRK family constitutes conserved serine/threonine kinases within the CMGC (including cyclin-dependent kinases (CDKs), mitogen-activated protein kinases (MAPKs), glycogen synthase kinases (GSKs), and CDK-like kinases) group and comprises five members: DYRK1A, –1B, −2, −3, and −4. DYRK1A is ubiquitously expressed in human tissues. In mouse and chick embryos, DYRK1A is mainly expressed in the central nerve system (CNS) and is involved in neurogenesis^2^. It also facilitates the progression of neural progenitor cells towards a more-differentiated state by inhibiting cell cycle progression^3^. Moreover, DYRK1A is correlated with synapse function by controlling synapse plasticity, pre- and post-synapse activities, and neurotransmission^4^.

The dysregulation of DYRK1A often leads to severe neurological disorders. The *DYRK1A* gene resides on the long arm of chromosome 21 at position 22.12 (21q22.12) and is identified in connection with Down syndrome (DS)^5^. DS is marked by an additional copy of chromosome 21, leading to heightened expression of genes located on this chromosome, including *DYRK1A*. Overexpression of DYRK1A is linked to intellectual disabilities and developmental delays, and it may contribute to the onset of early dementia in individuals with DS^6^. In contrast, heterozygous disruptions of the *DYRK1A* gene, including chromosome rearrangements, large deletions, and frameshifts of *DYRK1A,* can be classified as DYRK1A-haploinsufficiency syndrome. These patients present with microcephaly, intellectual disability, and development delays^6^. Together, these clinical observations imply that DYRK1A is critical for neural function.

Previous reports showed that DYRK1A is highly activated in the neocortex, entorhinal cortex, and hippocampus of Alzheimer’s disease (AD) patients^7^ and can phosphorylate downstream targets, including the amyloid precursor protein (APP) and tau protein, leading to the formation of amyloid β (Aβ) and neurofibrillary tangles (NFTs)^8^. Exploration of DYRK1A inhibitors has thus become a focal point of research attention in recent years. Inhibition of DYRK1A significantly decreased the formation of Aβ in APP/PS1 mice^9^. Moreover, levels of phosphorylated tau were markedly reduced by a DYRK1A inhibitor in JNPL3 tau mice^10^. Several inhibitors, such as DYR219^11^, L41^12^, and SM07883, demonstrated the potential to mitigate cognitive impairment in AD mice^10^. However, the major challenge of current inhibitors is their lack of selectivity, as some also inhibit other kinases within the CMGC family^13^. This off-target effect might induce unwanted outcomes, highlighting the importance of developing specific inhibitors for DYRK1A-related neurodegenerative diseases (NGDs).

**Figure 1.**
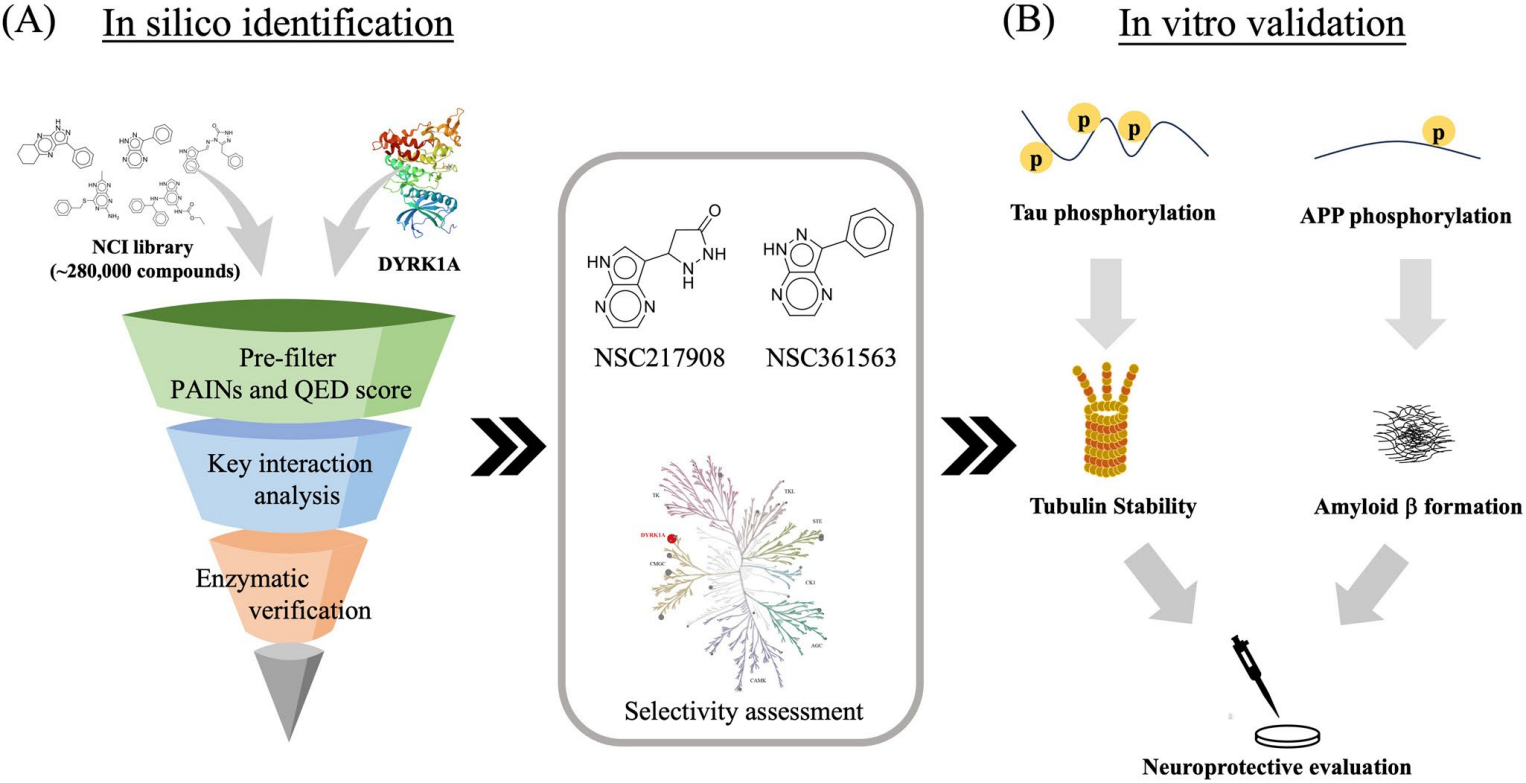
Schematic workflow for identifying DYRK1A inhibitors. (A) Strategy to select potential inhibitors targeting DYRK1A. (B) Potential DYRK1A inhibitors were further examined by *in vitro* functional assay. This figure was drawn by the authors.

Our aim in the present study was to identify novel DYRK1A inhibitors. We employed structure-based virtual screening (SBVS) to identify potential inhibitors of DYRK1A and then verified the efficacy of the compounds through *in vitro* validation (Figure 1). Approximately 280,000 compounds from the NCI library were docked to the DYKR1A-binding site, and top-ranked compounds were selected for further enzyme-based validation based on their docking scores and interactions. The most potent compound with high selectivity was then evaluated by an *in vitro* assay to evaluate its effects on reducing tau phosphorylation and increasing tubulin stability. Furthermore, the identified compound was found to decrease APP phosphorylation and Aβ formation, and reverse Aβ-induced neurotoxicity. Together, our research identified a novel DYRK1A inhibitor with therapeutic potential for AD.

## Materials and methods

### Molecular docking

Molecular docking was performed using the Maestro software suite by Schrödinger (https://newsite.schrodinger.com/platform/products/maestro/). The DYRK1A (PDB ID: 5A4E) protein structure was obtained from the public Protein Data Bank (https://www.rcsb.org) repository. The DYRK1A protein structure and compounds from the National Cancer Institute (NCI) screening library were respectively prepared using the Protein Preparation Wizard and LigPrep modules. Steps included removing water molecules, stripping salts from compounds, and generating relevant charges to the protein and compound structures. The co-crystal ligand was used to define the binding site and generate the receptor grid with default settings. Molecular docking was performed using the Glide module^14^. Protein-ligand interactions were generated using the “analyze non-bond interactions” component in Pipeline Pilot software^15^. Finally, figures for the protein-ligand docking were generated in 3D using PyMOL^16^.

### Protein kinase activity assay

To test the inhibitory effects of selected compound against potential kinases, the cell-free Z’-ZYTE^™^ kinase assay was used and conducted by ThermoFisher Scientific (www.thermofisher.com/selectscreen). Briefly, tested compounds, FRET (Fluorescence Resonance Energy Transfer) peptide/kinase mixture, and ATP were incubated at room temperature for 1 h. Development reagent was then added and incubated for another 1 h, and the kinase inhibitory activity was determined by the fluorescent signal ratio of 445 (coumarin)/520 nm (fluorescein) on a plate reader.

### Cell lines

The HEK-293 human embryonic kidney cell line was purchased from the Bioresource Collection and Research Centre (BCRC, Hsinchu, Taiwan) and was maintained in modified Eagle’s medium (MEM, ThermoFisher Scientific, Waltham, MA, USA) containing nonessential amino acids and supplemented with 10% (*v/v*) foetal bovine serum (FBS). The SH-SY5Y human neuroblastoma cell line was purchased from American Type Culture Collection (ATCC, Manassas, VA, USA) and cultured in a 1:1 mixture of Eagle’s minimum essential medium (EMEM) and F12 medium supplemented with 10% (*v/v*) FBS. All cells were incubated in 37 °C incubators containing 5% CO_2_

### Transient transfection

pEGFP-C1 human Tau cDNA encoding 441 amino acids was kindly provided by Dr. Chiung-Yuan Ko (School of Medicine, College of Medicine, National Sun Yat-Sen University, Kaohsiung, Taiwan). The mammalian expression plasmid for DYRK1A was constructed into a pDsRed-Monomer-Hyg-C1 vector (Clontech, CA, USA). The human APP tagged with GFP; cat. no. RG221339) was obtained from Origene (Rockville, MD, USA). For transfection, 1 µg of the selected plasmid in 125 µL Opti-MEM was gently mixed with 2 µL Lipofectamine^™^ 2000 (ThermoFisher Scientific) in 125 µL Opti-MEM. The mixture was incubated at room temperature for 20 min to form the DNA-lipid complex. This complex was then added dropwise onto 5 × 10^5^ cells and incubated at 37 °C for 24 h before proceeding with further experiments.

### Sample preparation and Western blot analysis

Cells (5 × 10^5^) were seeded in six-well plates overnight and then transfected with pDsRed-DYRK1A, pEGFP-Tau, or tGFP-APP. After 24 h, cells were treated with indicated concentrations of compounds for another 48 h. Sample lysate preparation and Western blotting were as previously described^17^. Briefly, protein samples were loaded onto a 10% SDS-PAGE gel and electrophoresed to separate proteins based on molecular weight. The proteins were then transferred from the gel to a PVDF membrane using a constant voltage of 400 mA for 120 min. The membrane was blocked with 5% milk in TBST (TBS with 0.1% Tween 20) for 1 h and then washed three times with TBST. Subsequently, the membrane was incubated with the indicated primary antibody at 4 °C overnight. After primary antibody incubation, the membrane was washed with TBST six times at 10-min intervals. The membrane was then incubated with an HRP-conjugated secondary antibody for 1 h. Following secondary antibody incubation, the membrane was washed again with TBST six times at 10-min intervals. The membrane was then incubated with a chemiluminescent substrate solution, and the signal was detected using an e-Blot imager (Shanghai, China). Primary antibodies of DYRK1A (#2771), Tau441 (#46687), Thr181-Tau (#12885), Thr217-Tau (#35834), and Thr668-APP (#6986) were purchased from Cell Signalling Technology (Danvers, MA, USA). Ser199/202-Tau (44–768 G) and Thr212-Tau (44–740 G) were obtained from ThermoFisher Scientific. The anti-APP antibody (Ab32136) was from Abcam (Cambridge, UK). The anti-β-amyloid, 1–16 antibody (803001) was obtained from Biolegend (CA, USA).

### Tubulin assembly assay

Polymerisation of tubulin protein was performed in a cell-free system according to the manufacturer’s instructions (BK006P, Cytoskeleton, Denver, CO, USA). Briefly, 11 µg of human recombinant Tau441 protein and 0.75 µg of DYRK1A protein were incubated in kinase buffer (20 mM MOPS pH 7.0, 10 mM MgCl_2_, 1 mM DTT, 20 mM sodium orthovanadate, and 0.3 mM ATP) at 30 °C for 2.5 h to generate phosphorylated tau (p-tau) protein. Levels of p-tau were determined by Western blotting.

Next, 3.7 µg recombinant tau and p-tau proteins were added to a preheated 37 °C 96-well plate containing 100 µg tubulin, 100 µL tubulin polymerisation buffer (comprising 750 µL Tubulin Buffer [80 mM PIPES pH 6.9, 2 mM MgCl_2_, 0.5 mM EGTA] and 250 µL Tubulin Glycerol Buffer [15% glycerol in General Tubulin Buffer]), and 10 µL of 100 mM guanosine 5′-triphosphate (GTP). After thorough mixing, polymerisation was measured at 340 nm using a spectrophotometer (Molecular Devices, Sunnyvale, CA, USA) every minute for a total of 45 min at 37 °C.

### Quantification of Aβ expression

To quantify the amount of Aβ, a specific Aβ42 ELISA kit (ThermoFisher Scientific, no. KHB3544) was employed. In brief, conditioned medium was collected and centrifuged at 1000 rpm for 3 min. The supernatant was then collected and added to a 96-well plate coated with the Aβ42 antibody, followed by overnight incubation at 4 °C. After washing four times, a secondary antibody was added for 30 min. Finally, a TMB-stabilized chromogen was utilised to visualise the reaction, and the absorbance was read at 450 nm.

### Oligomeric Aβ preparation

To prepare oligomeric Aβ, Aβ peptides (AS-24224, AnaSpec, CA, USA) were dissolved in DMSO and mixed with ice-cold serum-free F12 medium. These soluble monomeric Aβs were incubated at 4 °C for 24 h to yield oligomeric Aβ, which were confirmed by Western blotting.

### Cell viability and toxicity tests

Cell viability was assessed with a CCK8 assay by measuring dehydrogenase activity in living cells. In brief, 5 × 10^5^ cells were seeded in 96-well plates. Cells were incubated overnight and then supplemented with indicated concentrations of compounds or oligomeric Aβ for indicated times. Then the CCK-8 solution was added to each well for 1 h at 37 °C. The absorbance was measured at 450 nm by a microplate reader, and the cell survival rate was determined by dividing the value of each group by the value of the control group.

Cell toxicity was assessed with a lactate dehydrogenase (LDH) assay by measuring the LDH released from damaged cell membranes. Briefly, 5 × 10^5^ cells were seeded in 96-well plates. Cells were incubated overnight and then supplemented with indicated concentrations of compounds or oligomeric Aβ for indicated times. Following treatment, lysis buffer was applied to each well for 30 min at 37 °C, and a working solution was subsequently added for an additional 30 min. The reaction was stopped by the addition of a stop solution, and the absorbance was measured at 490 nm with a microplate reader.

## Ethics declarations

This study did not involve any human or animal subjects and therefore did not require ethical approval.

## Data analysis and statistics

All data were derived from three independent experiments, each with its own control group (vehicle without drug). The statistics were evaluated by comparing the dose-treated groups to their respective control groups within each experiment, and the control groups were normalised and presented as 100%. Western blot results were quantified using ImageJ software (National Institutes of Health, Bethesda, MD, USA) and analysed with GraphPad Prism software (GraphPad Software, San Diego, CA, USA). The intensity of the target proteins was first normalised to the corresponding housekeeping proteins and then further normalised to their respective total protein levels. The data are presented as the fold change relative to the control group, which was standardised to 100% in the figures. All data are presented as the mean ± standard deviation (SD) of at least three independent experiments. Parameters with *p* < 0.05 were considered statistically significant.

## Results

### Identification and validation of DYRK1A inhibitors

To identify potential DYRK1A inhibitors, we employed an SBVS campaign. Screened compounds were obtained from the NCI library, which consists of roughly 280,000 molecules. The library was first preprocessed before docking to reduce potential false-positive hits. This included the removal of compounds containing PAINS structures or having a qualitative estimate of drug-likeness (QED) score of <0.4^18^^,^^19^. Remaining compounds were molecularly docked into the DYRK1A-binding site. The resulting protein-compound poses were ranked based on their respective docking scores. The top-ranked compounds were selected and analysed for favourable interactions with DYRK1A. Small molecules targeting the kinase-binding site can be thought of as “ATP memetics”, and will generate distinct binding patterns with binding site residues^20^. Thus, hydrogen bonds to the kinase hinge region are important schemes for kinase inhibitors. Based on availability, 12 compounds were sourced for enzymatic assays. Two compounds, NSC217908 and NSC361563, showed respective 94 and 87% reductions in DYRK1A activity when tested at 10 µM (Table 1). Further investigations of the dose-dependent effects of these compounds revealed that NSC217908 had a 50% inhibitory concentration (IC_50_) of 948.74 nM, and NSC361563 had an IC_50_ of 1375.3 nM (Supplementary Figure 1). As a result, the SBVS identified two candidate DYRK1A inhibitors. These compounds served as candidates to test for potency and potential use in modulating tau phosphorylation.

**Table 1.** Enzymatic inhibitory activities of selected compounds.

Name	% Inhibition (10 μM)	Name	% Inhibition (10 μM)
217908 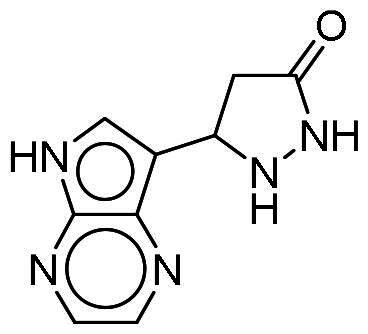	94	180969 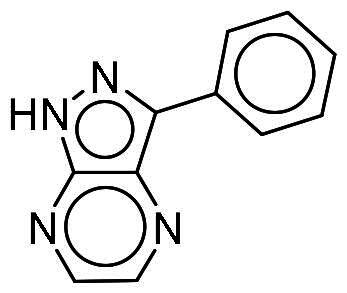	7
361563 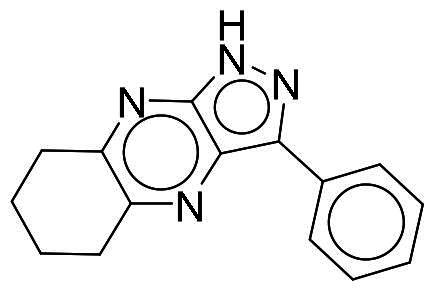	87	38731 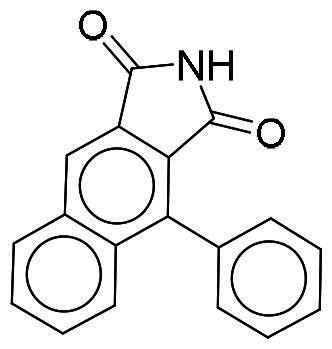	3
361564 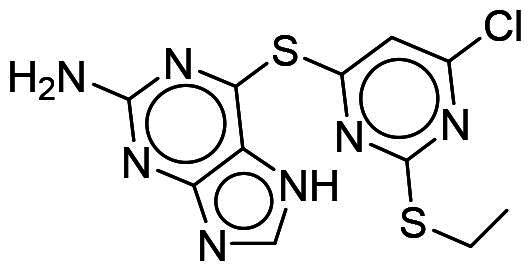	32	41663 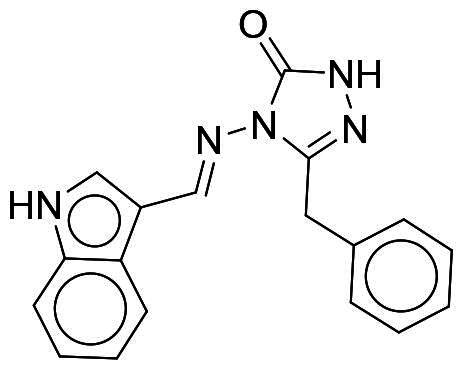	3
12102 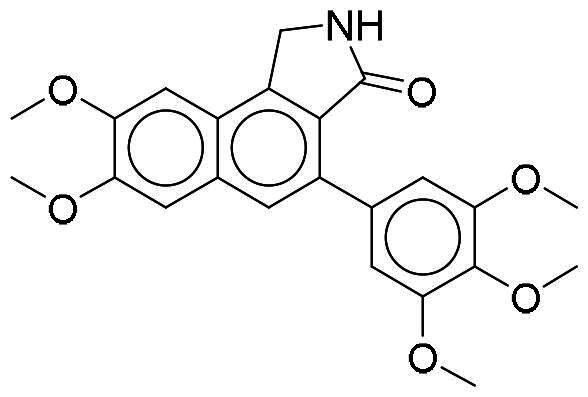	17	264867 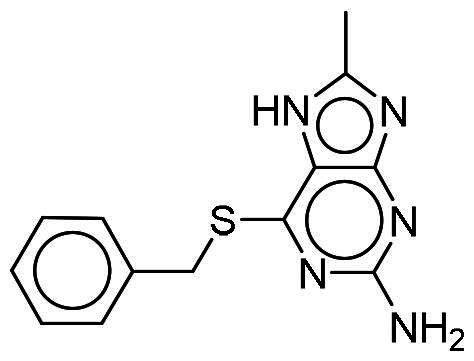	1
52385 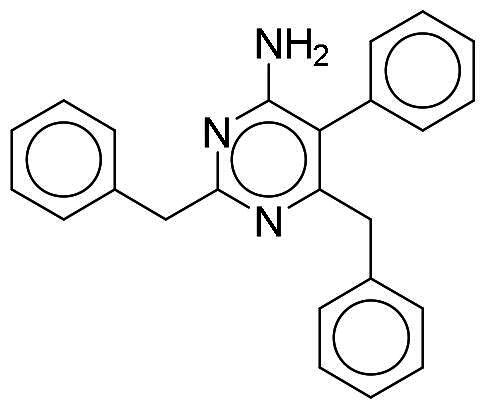	15	211688 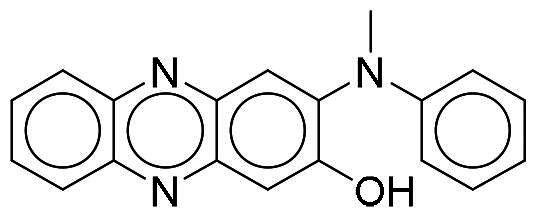	0
702401 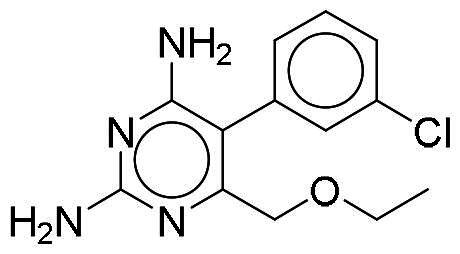	10	107390 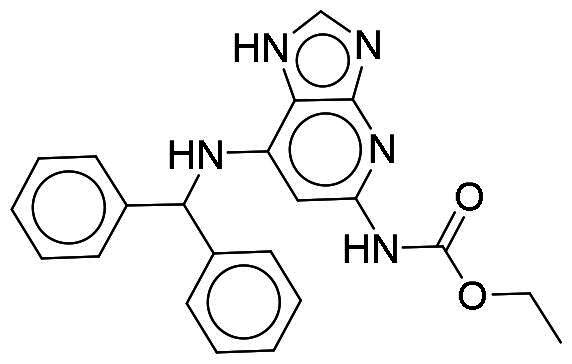	0

### Interaction analysis unveils favourable protein-ligand interactions

**Figure 2.**
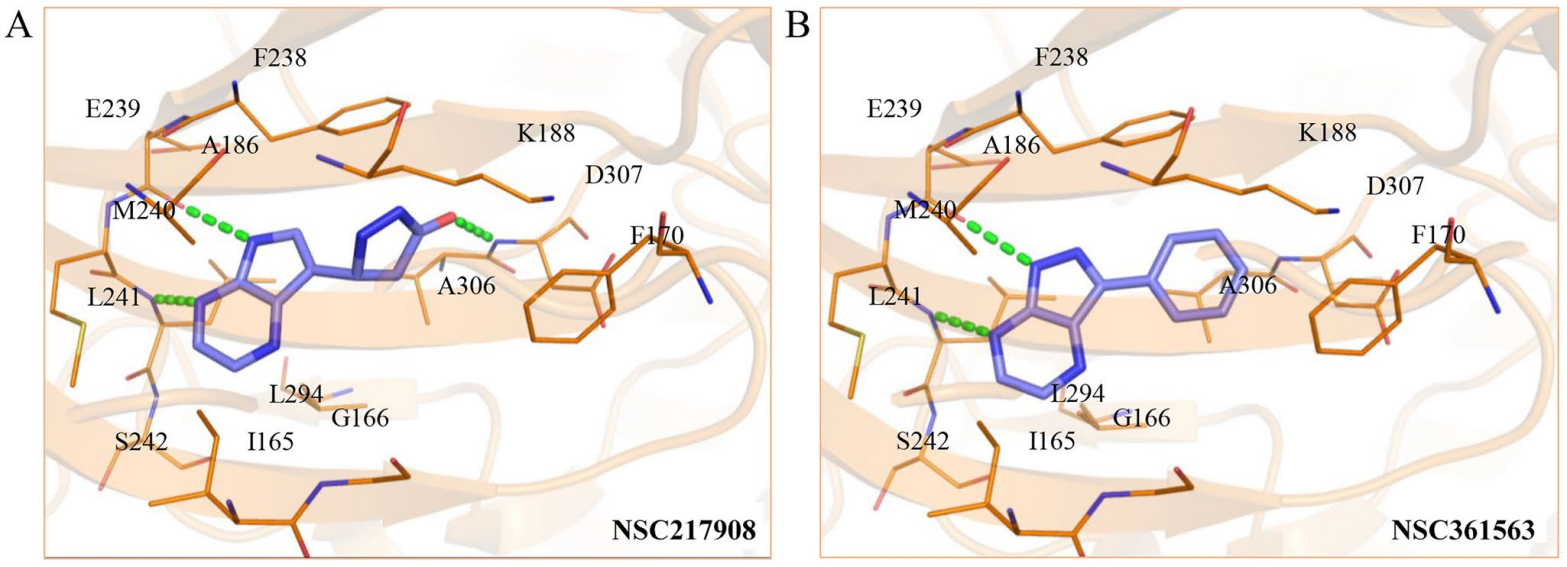
Interaction analysis of NSC217908 and NSC361563. Docking poses for (A) NSC217908 and (B) NSC361563 showed favourable occupation of the DYRK1A-binding site. This included hydrogen bonds to the DYRK1A hinge residue. The small molecules are rendered as sticks and in purple, while the DYRK1A-binding site is rendered in orange as a cartoon. Hydrogen bonds are denoted as green dashes. Binding site residues are rendered as lines and labelled as shown.

From our selection, two compounds, NSC217908 and NSC361563, displayed the most potent inhibitory activities. Their docking poses shows favourable positioning within the DYRK1A-binding site. In particular, the compounds form two hydrogen bonds to hinge residues E239 and L241. NSC217908 produces an additional hydrogen bond with residue D307. This is due to the hydrogen acceptor on the pyrazolidin‐3‐one moiety present on NSC217908 (Figure 2(A)). In contrast, NSC361563 contains a benzyl moiety, which would not facilitate traditional hydrogen bond interactions (Figure 2(B)). This additional hydrogen bond contributes to the observed differences in the inhibitory effectiveness, as evidenced by the lower IC_50_ value of NSC217908 compared to NSC361563, highlighting its superior inhibitory potency (Supplementary Figure 1). The kinase-binding site typically contains hydrophobic residues that would sandwich the adenosine scaffold of ATP^20^. This is also observed for NSC217908 and NSC361563, with residues A186, L241, L294, and V306 contributing to hydrophobic interactions. Interestingly, these interactions were identified as key pharmacological interactions for DYRK1A inhibitors^21^. Thus, interactions by both NSC217908 and NSC361563 would suggest favourable binding within the DYRK1A-binding site. Additionally, hydrophobic interactions between M240 and F238 were respectively observed for NSC217908 and NSC361563. To validate interactions of NSC217908 and NSC361563 with the DYRK1A ATP-binding site, experiments were conducted using various ATP concentrations with 3 μM of each compound. The findings demonstrated that as the ATP concentration increased, the inhibitory efficacy of both NSC217908 and NSC361563 decreased in a dose-dependent manner, confirming their mechanism of action as competitive ATP inhibitors (Table 2). Slight interaction profile differences between the two compounds may have been due to their spatial positioning in the DYRK1A-binding site. In particular, the pyrazolidin‐3‐one moiety for NSC217908 is angled almost 90° compared to the benzyl moiety of NSC361563 (Figure 2(B)). Altogether, the interaction profile suggested favourable occupation of and possible occlusion of the DYRK1A-binding site.

**Table 2.** Inhibitory activities of NSC217908 and NSC361563 at 3 μM using different doses of ATP.

Compound	ATP (µM)	Inhibition (%)	Compound	ATP (µM)	Inhibition (%)
NSC217908	1000	62	NSC361563	1000	17
NSC217908	100	57	NSC361563	100	67
NSC217908	10	97	NSC361563	10	72

### Selectivity of NSC217908 and NSC361563

**Figure 3.**
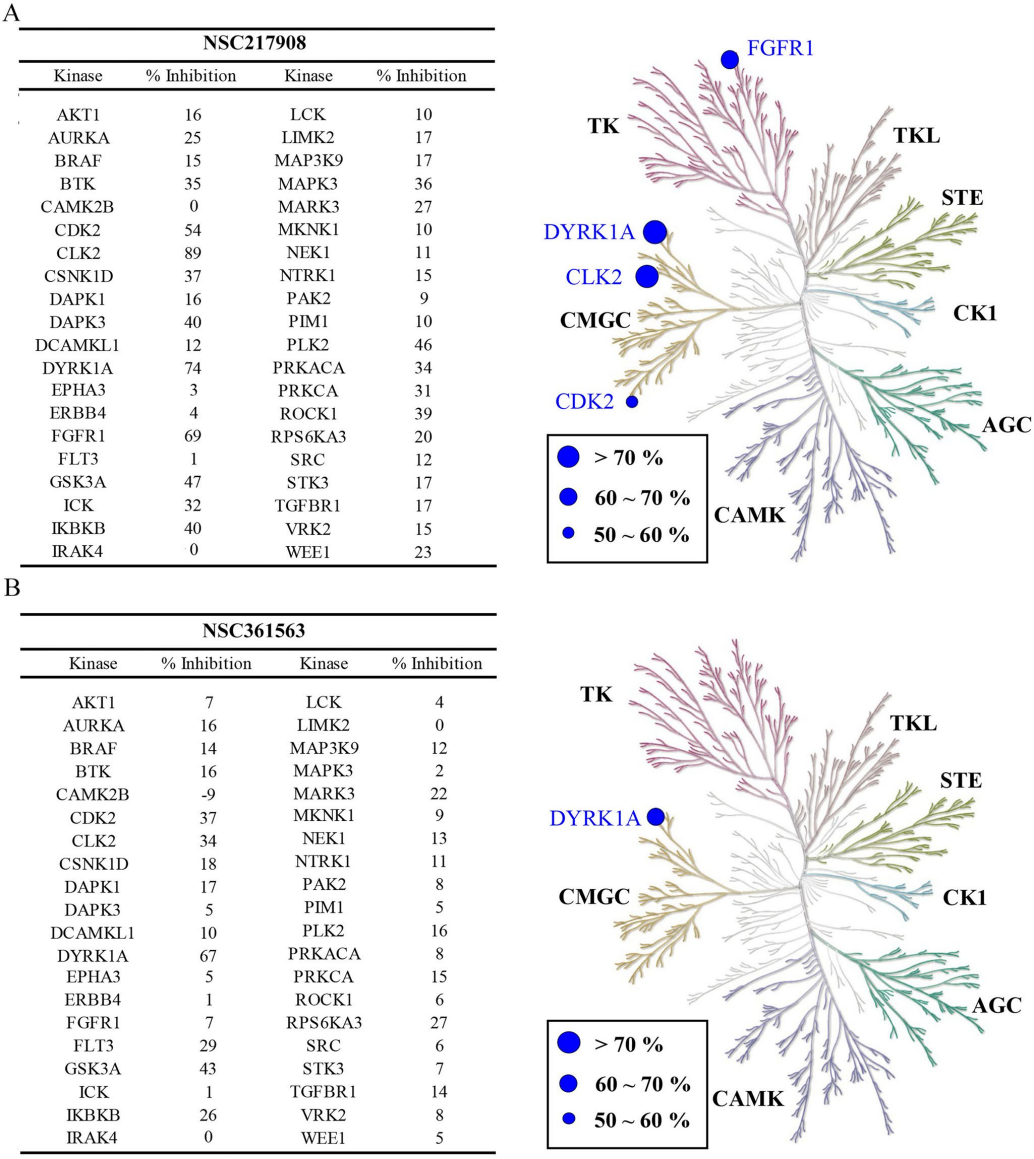
Selectivity profile of DYRK1A inhibitors across the human kinome. Inhibitory activities of (A) NSC217908 and (B) NSC361563 were assessed at a concentration of 3 μM across a panel of 40 protein kinases. Inhibitory effects above 50% are indicated by blue dots.

Considering the high homology among the catalytic domains of human protein kinases, inhibitors designed to target the ATP-binding domain often inadvertently affect multiple kinases, which could lead to potential unwanted side effects. To evaluate the selectivity of NSC217908 and NSC361563, comprehensive screening across a panel of 40 human protein kinases was conducted. Results indicated that NSC217908 exhibited a broad targeting spectrum, primarily affecting kinases within the CMGC family, including significant inhibition of DYRK1A (74%), cyclin-dependent kinase 2 (CDK2; 54%), and CDC-like kinase 2 (CLK2; 89%) at 3 μM. Additionally, NSC217908 inhibited kinases outside of the CMGC family, such as fibroblast growth factor receptor 1 (FGFR1; 69%) (Figure 3(A)). In comparison, NSC361563 demonstrated higher specificity towards DYRK1A, with it being the only kinase showing more than 50% inhibition by NSC361563 at 3 μM (Figure 3(B)), highlighting its selective inhibitory profile.

### In vitro evaluation of NSC361563 and NSC217908 on DYRK1A-induced tau phosphorylation

**Figure 4.**
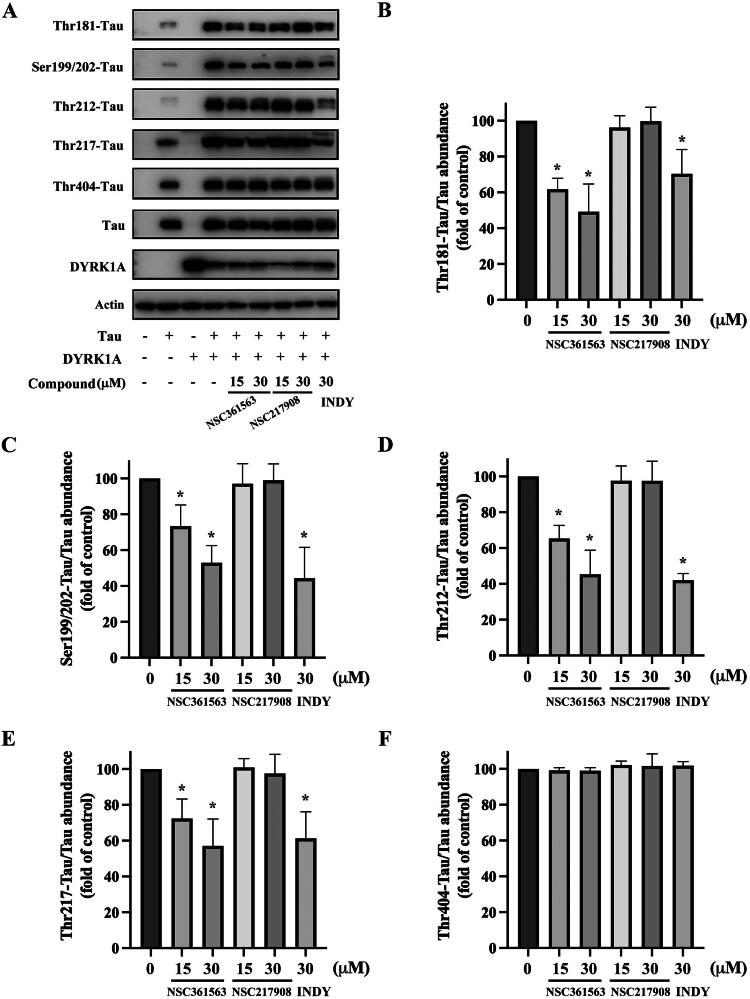
Effects of NSC361563 and NSC217908 on DYRK1A-induced tau phosphorylation. (A) HEK293 cells were transfected with DYRK1A, tau, or their combination for 24 h, and then treated with indicated concentrations of compounds for another 48 h. Phosphorylation levels of specific tau were measured by Western blotting. Quantitative results of (B) Thr181-tau, (C) Ser199/202-tau, (D) Thr212-tau, (E) Thr217-tau, and (F) Thr404-tau were calculated using ImageJ software, based on data from at least three independent experiments. *Compared to the DYRK1A and tau co-expression group.

Previous research identified DYRK1A as one of the upstream kinases involved in regulating tau phosphorylation^22^. To investigate the impacts of NSC217908 and NSC361563 on DYRK1A activity, we established a cell line using HEK293 cells that were transfected with DYRK1A, full-length tau441, or their combination. Our results showed that DYRK1A expression alone did not alter the phosphorylation status of tau. In contrast, cells expressing tau demonstrated an increase in tau phosphorylation at multiple sites, including Ser199/202, Thr181, Thr212, Thr217, and Ser404. Interestingly, co-expression of DYRK1A and tau led to further elevation of phosphorylation at all of these sites except for Ser404. This observation emphasised the appropriateness of this model for assessing the effectiveness of NSC217908 and NSC361563 in targeting DYRK1A. Results indicated that NSC361563 dose-dependently reduced tau phosphorylation levels at Ser199/202, Thr181, Thr212, and Thr217 (Figure 4(A)). Although NSC217908 had superior inhibitory activity in enzymatic assays targeting DYRK1A, it showed no effect on tau phosphorylation. As a positive control, we included INDY, a potent DYRK1A inhibitor with an IC_50_ value of 240 nM, which also reduced tau phosphorylation at specified sites. Although NSC361563 possessed a higher IC_50_ compared to INDY, it demonstrated comparable effects in reducing tau phosphorylation at Ser199/202, Thr212, and Thr217. Moreover, NSC361563 demonstrated a potentially superior inhibitory effect at Thr181, highlighting its efficacy in regulating tau phosphorylation via DYRK1A inhibition (Figure 4(B–F)).

### Tubulin stability evaluation of NSC361563

**Figure 5.**
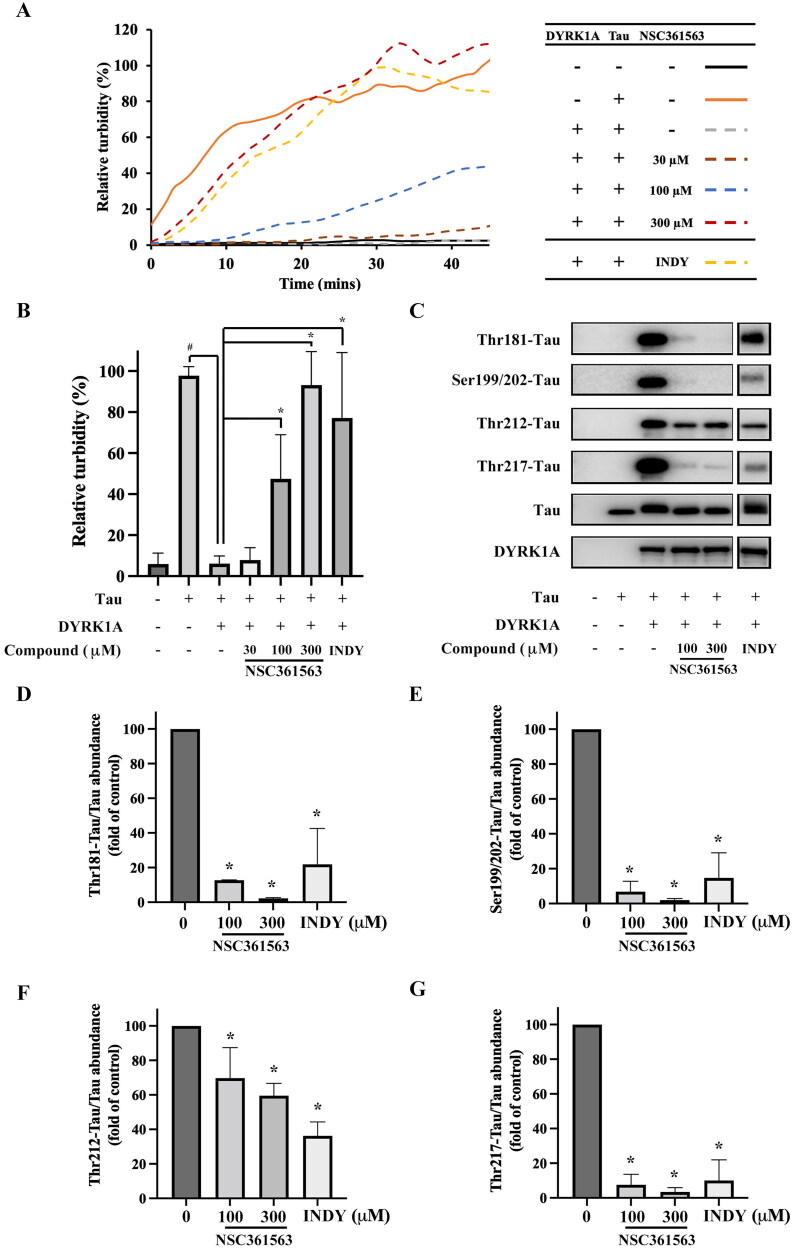
Effect of NSC361563 on tubulin dynamics. (A) Recombinant DYRK1A and tau were added to solutions containing tubulin, tubulin polymerisation buffer, and GTP. Tubulin polymerisation was measured by the solution turbidity using a spectrophotometer. (B) Quantitative results of NSC361563 on tubulin polymerisation. ^#^Compared to the tau alone group. *Compared to the combined DYRK1A and tau group. (C) Levels of tau phosphorylation were measured by Western blotting. Quantitative results of (D) Thr181-tau, (E) Ser199/202-tau, (F) Thr212-tau, and (G) Thr217-tau were calculated using ImageJ software, based on data from at least three independent experiments. *Compared to the combined DYRK1A and tau group.

One of the functions of tau is to regulate microtubule stability and maintain intracellular transport and cell plasticity. In its normal state, the tau protein binds to microtubules and promotes their assembly, contributing to their structural integrity. However, when tau becomes hyperphosphorylated, its affinity for microtubules decreases, leading to disruption in their dynamic behaviour^23^. Since NSC361563 reversed DYRK1A-induced tau phosphorylation, we further tested its impact on tubulin stability. In a cell-free assay, the tau protein alone exhibited the ability to stabilise tubulin, promoting tubulin assembly and resulting in an increase in turbidity over time. Conversely, when DYRK1A and tau were simultaneously introduced into the reaction, tubulin underwent depolymerisation, leading to a decrease in turbidity. NSC361563 dose-dependently increased turbidity and promoted tubulin assembly (Figure 5(A,B)). A Western blot analysis further validated tau phosphorylation levels, demonstrating that NSC361563 effectively inhibited DYRK1A activity, leading to reductions in tau phosphorylation at Ser199/202, Thr181, Thr212, and Thr217 (Figure 5(B–F)). These results were consistent with our findings in the *in vitro* model (Figure 4(A)). Furthermore, the cell-free system only included DYRK1A, tau, tubulin, and NSC361563, eliminating the possibility of NSC361563 acting via alternative mechanisms; therefore, the results suggested its role as a direct DYRK1A inhibitor that impacts tau phosphorylation. Collectively, these findings suggest that NSC361563 not only inhibited the effect of DYRK1A on tau phosphorylation but also contributed to further stabilisation of tubulin assembly.

### In vitro evaluation of NSC361563 and NSC217908 on DYRK1A-induced APP phosphorylation

**Figure 6.**
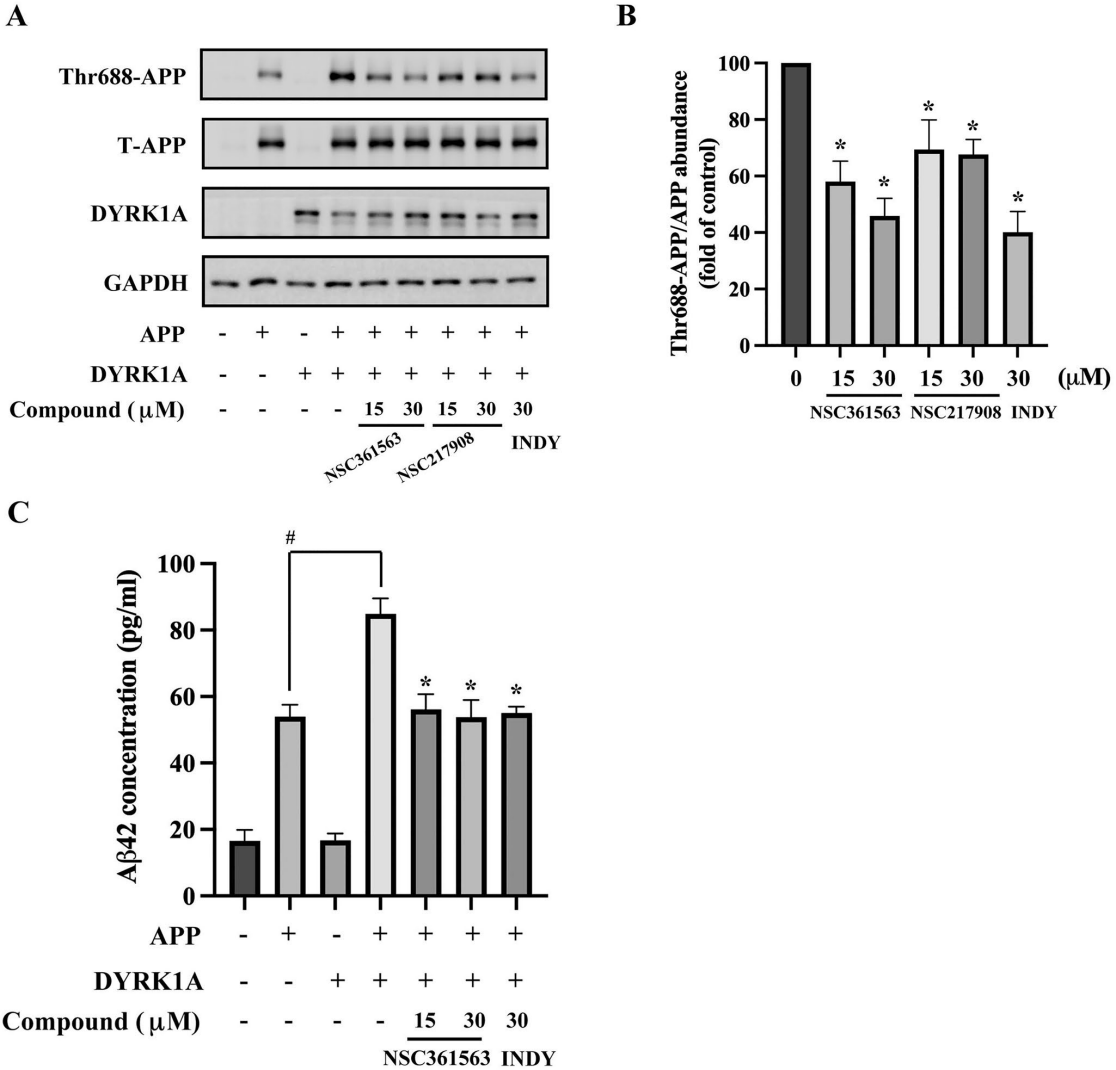
Impacts of NSC361563 and NSC217908 on amyloid β (Aβ) formation. (A) HEK293 cells were transfected with DYRK1A, amyloid precursor protein (APP), or their combination for 24 h, and then treated with indicated concentrations of compounds for another 48 h. Phosphorylation levels of Thr699-APP were measured by Western blotting. (B) Quantitative results were measured by ImageJ software, based on data from at least three independent experiments. *Compared to the DYRK1A and tau co-expression group. (C) HEK293 cells were transfected with DYRK1A, APP, or their combination for 24 h, and then treated with indicated concentrations of compounds for another 48 h. Conditioned medium was collected and then analysed with an Aβ42 ELISA kit. ^#^Compared to the APP alone group. *Compared to the combined DYRK1A and APP group. Data from at least three independent experiments were analysed.

Excessive levels of Aβ have been identified in the hippocampus and cortex of individuals with AD^24^. This peptide is generated through the proteolytic processing of the APP. Herein, we demonstrated that cells transfected with APP showed a slight increase in phosphorylation at Thr668, which was further amplified in the presence of DYRK1A (Figure 6(A)). NSC361563 was observed to reduce APP phosphorylation at Thr668 in a dose-dependent manner, achieving 45% reduction at 30 μM. NSC217908 exhibited a less-significant effect on APP phosphorylation, with a 67% reduction at the same concentration (Figure 6(B)). To assess the impact of NSC361563 on Aβ formation, conditioned medium from cells co-expressing DYRK1A and APP was analysed using an Aβ42 ELISA kit. Consistent with Thr688-APP levels, levels of Aβ42 increased in the presence of both DYRK1A and APP compared to APP alone. Notably, this phenomenon could be reversed by NSC361563 treatment (Figure 6(C)). Unlike tau, which has multiple DYRK1A-mediated phosphorylation sites, Thr668 is currently the only known DYRK1A-mediated site on APP. This specificity was reflected in the maximal 54% reduction in Aβ42 formation observed with a 30 μM dose of NSC361563. Together, our findings demonstrated that NSC361563 effectively reduced APP phosphorylation and Aβ42 production.

### Investigation of the neuroprotective effect of NSC361563 against aβ-induced cell death

**Figure 7.**
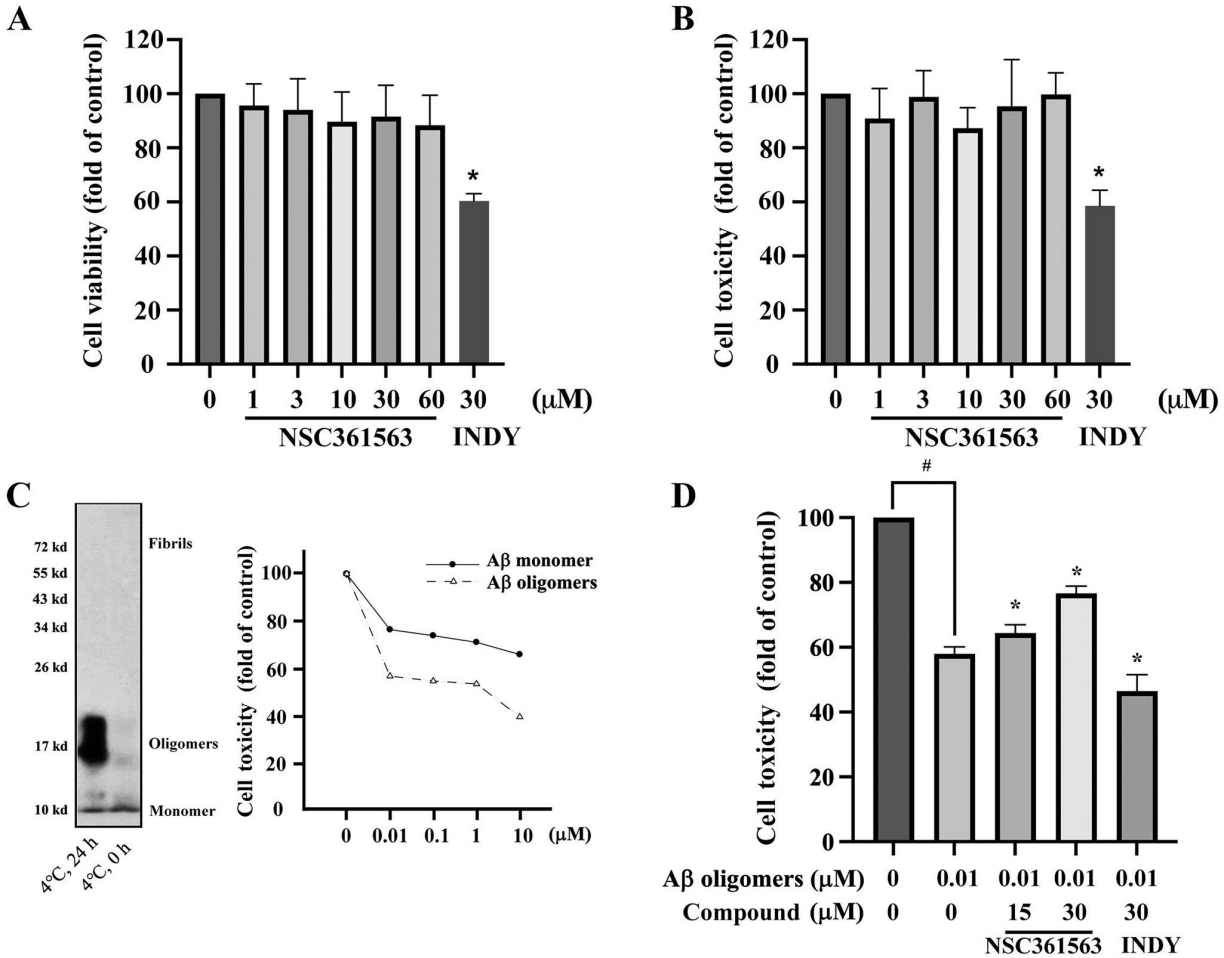
Effects of NSC361563 on neuronal cell viability and toxicity. (A) Cell viability was assessed using a CCK8 assay following treatment with various concentrations of NSC361563 for 48 h. (B) Cell toxicity was evaluated using a lactate dehydrogenase (LDH) assay after treating cells with different doses of NSC361563 for 48 h. (C) SH-SY5Y cells were exposed to monomeric or oligomeric amyloid β (Aβ) for 48 h, and cell toxicity was measured using an LDH assay. (D) Cells were pre-treated with NSC361563 or INDY for 30 min before the addition of 0.01 μM oligomeric Aβ for an additional 48 h, with cell toxicity assessed using an LDH assay. ^#^Compared to the control group without oligomeric Aβ or treatment compounds. *Compared to the 0.01 µM oligomeric Aβ group. Data from at least three independent experiments were analysed.

Given their pivotal roles in regulating body functions and their limited regenerative ability, neuronal cells are irreplaceable. Therefore, it is essential that the safety of drugs designed to treat NGDs is prioritised. To address this issue, we conducted cell-viability and cell-toxicity assays. Results showed that NSC361563 at doses of <60 µM induced no changes in cell proliferation or cell death (Figure 7(A,B)). This dose is considerably higher than the dosage we used to inhibit DYRK1A activity in previous experiments. Aβ aggregation is associated with neuronal cell death in AD, particularly with the soluble form of the Aβ oligomer which is deemed highly toxic. In our study, we successfully obtained the Aβ oligomer from the Aβ monomer (Figure 7(C)), and further tested their cytotoxicity against human neuroblastoma SH-SY5Y cells. Results showed that the oligomeric Aβ significantly induced cell death at a concentration of 0.01 μM in the absence of FBS (Figure 7(D)). Pretreating cells with NSC361563 for 30 min prior to the addition of oligomeric Aβ for 48 h showed that NSC361563 dose-dependently reduced neuronal cell death, reversing the effects of oligomeric Aβ. On the contrary, INDY was not only ineffective in providing neuroprotection against oligomeric Aβ but also exhibited cytotoxicity when it alone was used to treat neuronal cells. This adverse effect of INDY might be attributed to its lack of selectivity, potentially inhibiting other CMCG family members like CDK, CLK, creatine kinase (CK), and glycogen synthase kinase 3 (GSK3)^25^. These results suggested that NSC361563 reversed Aβ oligomer-induced neuronal cell death and may be considered a potential neuroprotective agent which should be further evaluated.

## Discussion

DYRK1A plays a crucial role in regulating numerous physiological functions, with cognitive functions being the most extensively studied. Dysregulation of DYRK1A often leads to severe NGDs, such as Parkinson’s disease, DS, and AD, indicating that targeting DYRK1A for drug design holds great potential. Given its significance in neurodegenerative diseases, an increasing number of structurally diverse inhibitors have been discovered, some demonstrating nanomolar potency. However, achieving selectivity remains a major challenge for these compounds^26^. In the present study, we employed SBVS to identify DYRK1A inhibitors. The compound NSC361563 emerged as being capable of inhibiting DYRK1A activity with high selectivity. Additionally, it demonstrated potential in stabilising tubulin, diminishing Aβ production, and offering neuroprotection, suggesting its therapeutic potential for AD.

Our previous study highlighted key residues within the ATP-binding pocket of DYRK1A, with L241 forming part of the hinge loop^21^. In the present study, we showed that NCS217908 with the core structure of 5H-pyrrolo[2,3-b]pyrazine, and NSC361563 with the core structure of 2H-pyrazolo[3,4-b]pyrazine, forms hydrogen bonds with the E239 and L241 DYRK1A hinge residues. Moreover, hydrophobic interactions of A186, L241, L294, and V306 further stabilise the core scaffolds of these compounds within the DYRK1A active site. With an additional hydrogen bond at residue D307, NSC217908 exhibited stronger inhibitory activity against DYRK1A than did NSC361563 (Figure 2(A,B)). The kinase profile showed that both NCS217908 and NSC361563 inhibited DYRK1A activity, with NCS217908 also targeting other kinases such as CDK2, CLK2, and FGFR1 (Figure 3). Previous studies showed that FGF10 treatment reduced tau phosphorylation^27^, and FGF21 alleviated Aβ- and tau-induced pathology in an AD rat model^28^. Therefore, the interaction of FGFR1 with NSC217908 may account for the lower efficacy in reducing tau phosphorylation in the assays (Figures 4(A) and 5(A)). On the other hand, NSC361563 exhibited a highly selective profile towards DYRK1A, showing minimal effects on other CMGC family members like CDK and CLK (Figure 3). The selectivity of NSC361563 may result from its interactions with non-conserved residues. To investigate this, we conducted a conservation analysis of the DYRK1A binding site. Binding site sequences from various kinases were obtained from the KLIFS database (Kinase–Ligand Interaction Fingerprints and Structures, https://klifs.net/), which defines 85 aligned binding site residues (Supplementary Figure 2(A)). These aligned residues were then submitted to the WebLogo server (https://weblogo.berkeley.edu/logo.cgi) to generate sequence logos representing conservation levels (Supplementary Figure 2(B)). Comparison of the interacting residues of NSC361563 revealed three key non-conserved residues contributing to its selectivity: V306 (KLIFS position: 80), F238 (KLIFS position: 45), and L241 (KLIFS position: 48) (Supplementary Figure 2(C)). Among these, F238 is the gatekeeper, playing a crucial role in substrate binding and inhibitor selectivity^29^. The analysis suggests that hydrophobic interactions with the non-conserved residues V306, F238, and L241 may underlie the selectivity of NSC361563. This selectivity is crucial, as most current DYRK1A inhibitors, such as EGCG, harmine, lamerallins, and meriolins, exhibit non-specific inhibition of other kinases, particularly those within the CMGC family^30^. Such off-target effects might cause unwanted side effects, with reports suggesting that inhibiting CDK5 activity may lead to bone marrow suppression^31^, and suppressing CLK could affect RNA splicing, which is associated with a range of complex cellular outcomes^32^. Therefore, the high selectivity of NSC361563 makes it an ideal candidate for studying the effects of DYRK1A inhibition.

The structure of full-length tau contains 441 amino acids, which can be divided into a projection domain and a microtubule assembly domain. In our study, NSC361563 bound to the DYRK1A-binding site and strongly inhibited tau phosphorylation at multiple sites, including Ser199/202, Thr181, Thr212, and Thr217 (Figure 4(A)). These binding sites are located in the microtubule assembly domain of the tau protein, while adding a phospho-group may alter the local charge effect and interfere with the interaction of tau and microtubules. Therefore, NSC361563 also effectively caused tubulin polymerisation by reducing tau phosphorylation caused by DYRK1A (Figure 5(A)). Clinically, phosphorylation of Thr181 and Thr217 is thought to be biomarkers of early AD^33^. Phosphorylation of Ser199/202 and Thr212 may contribute to tau self-assembly and ultimately the formation of NFTs^34^. Importantly, these phosphorylation sites do not function independently but interact with each other, suggesting that the interplay among various phosphorylation events can exacerbate the tau-related pathogenesis. Our research revealed that NCS361563 can inhibit multiple tau phosphorylation sites, thereby markedly promoting tubulin stability through inhibiting DYRK1A activity.

Aggregated Aβ is considered neurotoxic, leading to irreversible damage to and dysfunction of neuronal cells. Our study demonstrated that both NSC361563 and NSC271908 decreased APP phosphorylation, with NSC361563 showing superior potency (Figure 6(A)). The APP undergoes several cleavage processes, including β-secretase and γ-secretase activities, resulting in the production of APP fragments that further aggregate into insoluble Aβ^35^. Phosphorylation at Thr668 is known to enhance APP cleavage by β-secretase^36^. Our data also revealed that the Aβ42 level decreased following treatment with NSC361563 (Figure 6(C)). To fully explore the effect of NSC361563 on Aβ toxicity, we further showed that exogenous administration of oligomeric Aβ induced neuronal cell death, which could be reversed by NSC361563 treatment (Figure 7(C,D)). Abundantly expressed Aβ exhibits prion-like characteristics and exerts toxicity towards neuronal cells through upregulating the inflammatory response^37^ and oxidative stress^38^. A previous study showed that DYRK1A inhibition reduced the production of proinflammatory cytokines via STAT3 dephosphorylation^9^. Additionally, DYRK1A was implicated in oxidative stress during LPS-induced neuroinflammation^39^. Therefore, the neuroprotective effects of NSC361563 may be attributed to its ability to inhibit inflammatory and oxidative responses.

Over the past few decades, many drugs designed to alleviate AD have demonstrated efficacy in animal studies but encountered failure in human clinical trials. One of the reasons is the inability of those drugs to penetrate the blood-brain barrier (BBB). The BBB is composed of selective semipermeable endothelial cells that protect our brains from foreign invaders. However, this protective nature also poses a challenge by impeding the effectiveness of drugs intended to treat CNS diseases^40^. In the past, a significant portion of drugs entering clinical trials were designed as antibodies targeting Aβ. While antibody drugs have shown effectiveness, they also face the drawback of having a large molecular structure, making it challenging to penetrate the BBB. In contrast, small-molecule drugs, due to their relatively small structure, have a higher probability of crossing the BBB. To assess compound ADMET (adsorption, distribution, metabolism, excretion, and toxicity) properties, particularly BBB penetration, we employed ADMETlab 2.0 (https://admetmesh.scbdd.com/)^41^. The Caco-2 permeability score indicated that NSC361563 (-4.769) was comparable to the clinical drug donepezil (-4.793). Moreover, NSC361563 exhibited a good BBB-penetration ability. To predict the toxicity profile, we utilised ProTox-II (https://tox-new.charite.de/protox_II/)^42^. Results categorised both NSC361563 and donepezil as class IV, with respective LD_50_ values of 1000 and 505 mg/kg, while INDY exhibited a value of 370 mg/kg (Supplementary Table 1). This toxicity prediction is consistent with our *in vitro* results (Figure 7), suggesting that NSC361563 may be a safe candidate for AD treatment. Future studies will focus on verifying these pharmacokinetic and toxicity profiles of NSC361563 through rigorous in vivo testing.

Given the significant societal and economic impacts of AD, there is a pressing demand for the development of therapeutic drugs. Simultaneously targeting both the production of Aβ and tau phosphorylation may achieve enhanced therapeutic outcomes. In this study, we reported a novel DYRK1A inhibitor using the SBVS approach and identified NSC361563 and NSC271908 as compounds with multiple interactions within DYRK1A-binding sites. Moreover, NSC361563 exhibited a selective profile towards DYRK1A. Inhibition of DYRK1A by NSC361563 not only inhibited tau protein phosphorylation but also promoted tubulin polymerisation, further enhancing tubulin stability. On the other hand, NSC361563 also reduced APP phosphorylation and decreased Aβ42 production. Notably, NSC361563 exhibited neuroprotective properties, successfully reversing Aβ-induced neurotoxicity. Our previous study has identified the pharmacological interactions of DYRK1A using known DYRK1A inhibitors, which may serve as a hint for future optimisation. The pharmacological interactions include two hydrogen-bonding (LYS188 and LEU241) and seven hydrophobic interactions (VAL306, LYS188, ALA186, VAL173, PHE238, LEU294, and LEU241)^21^. The docking results of NSC217908 and NSC361563 revealed that both compounds form one pharmacological hydrogen bond and four pharmacological hydrophobic interactions (Supplementary Figure 3). Analysis of the pharmacological interactions suggested that adding additional moieties to both compounds to form the hydrogen bond with residue LYS188 and the hydrophobic interactions with LYS188, VAL173, and PHE238 could improve their activity. According to previous studies^43^, the salt bridge formed with LYS188 helps to stabilise the binding of the ligands in the ATP-binding site, contributing to the overall strength of their interaction with DYRK1A. These analysis can serve as a guide to improve the activity of the identified hits. In summary, our data provide a therapeutic opportunity of NSC361563 for further optimisation in neurodegenerative diseases.

## Supplementary Material

Supplemental Material

Supplemental Material

## Data Availability

The datasets presented in the current study are available from the corresponding author upon reasonable request.
