# Supplementary material for: Discovering a novel dual specificity tyrosine-phosphorylation-regulated kinase 1A (DYRK1A) inhibitor and its impact on tau phosphorylation and amyloid-β formation
Source: J Enzyme Inhib Med Chem. 2024 Nov 4;39(1):2418470. doi: 10.1080/14756366.2024.2418470 (PMC11536634; doi:10.1080/14756366.2024.2418470)
Supplement: Supplemental Material [file IENZ_A_2418470_SM5532.pdf]

Supplementary Table 1. Predicted Caco-2 permeability, blood-brain barrier (BBB) penetration, and toxicity of various compounds

Compound	Caco-2 permeability	BBB penetration	Toxicity (LD_{50})
NSC361563	-4.769	good	1000 mg/kg
INDY	-4.534	excellent	370 mg/kg
Donepezil	-4.793	poor	505 mg/kg

LD_{50}, 50% lethal dose
