# Supplementary material for: Discovering a novel dual specificity tyrosine-phosphorylation-regulated kinase 1A (DYRK1A) inhibitor and its impact on tau phosphorylation and amyloid-β formation
Source: J Enzyme Inhib Med Chem. 2024 Nov 4;39(1):2418470. doi: 10.1080/14756366.2024.2418470 (PMC11536634; doi:10.1080/14756366.2024.2418470)
Supplement: Supplemental Material [file IENZ_A_2418470_SM5525.pdf]

Supplementary Figures

A

Compound	Concentration (nM)	Inhibition (%)
NSC217908	10000	94
NSC217908	3000	74
NSC217908	1000	50
NSC217908	300	27
NSC217908	100	8

Compound	Concentration (nM)	Inhibition (%)
NSC361563	10000	87
NSC361563	3000	67
NSC361563	1000	41
NSC361563	300	17
NSC361563	100	7

B

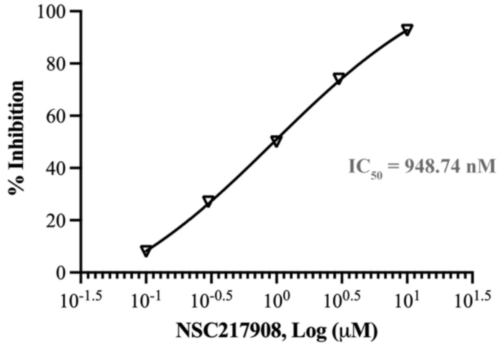

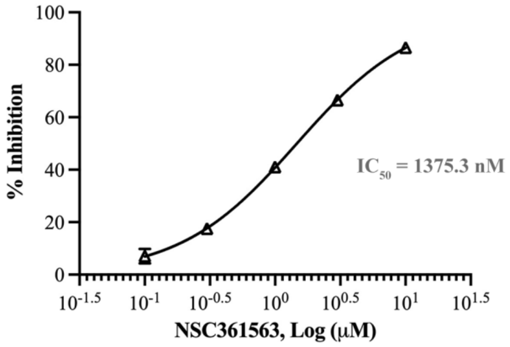

Supplementary Figure 1. Inhibitory effects of NSC217908 and NSC361563 against DYRK1A. (A) The inhibitory effects of selected compounds at different concentrations. (B) The dose-response curves of selected compounds against DYRK1A.

A

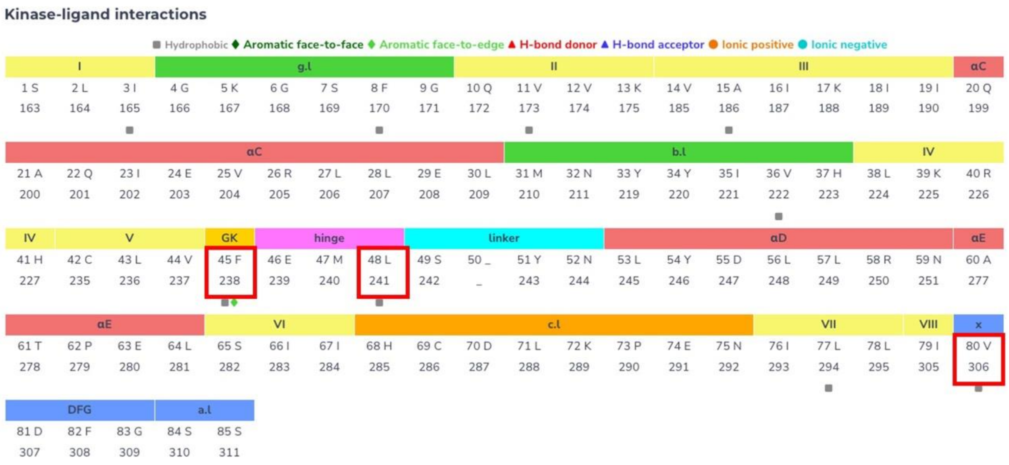

B

Pharmacological interactions	Hydrogen Bond		Hydrophobic				
NSC361563	GLU239	LEU241	ALA186	PHE238	LEU241	LEU294	VAL306
KLIFS nomenclature	46	48	15	45	48	77	80

C

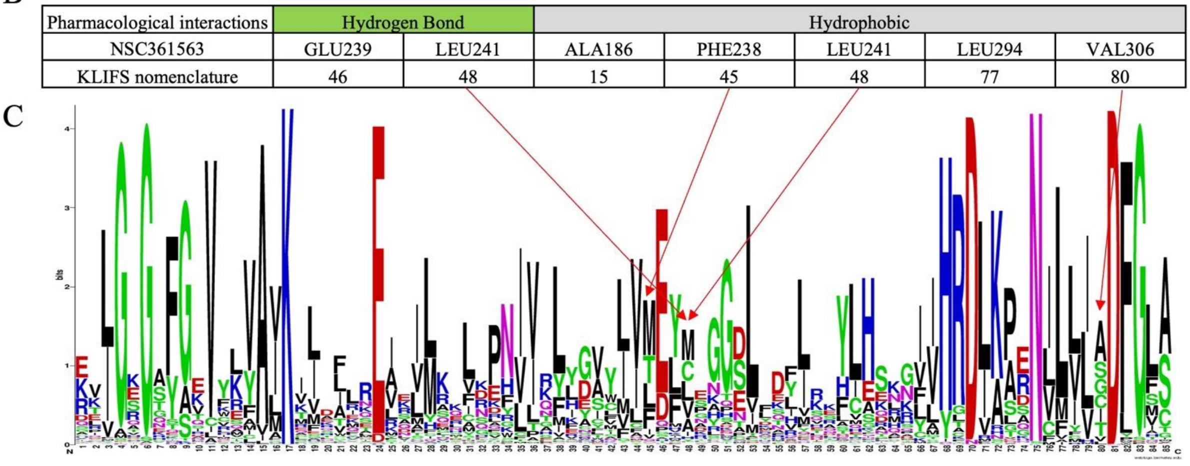

Supplementary Figure 2. Conservation analysis of NSC361563 interacting residues. (A) Residue numbering of the DYRK1A binding site according to the KLIFS definition. (B) Interactions between the DYRK1A binding site and NSC361563. (C) Non-conserved residues interacting with NSC361563.

Pharmacological interactions		Hydrogen Bond		Hydrophobic Bond						
Residue		LYS188	LEU241	VAL306	LYS188	ALA186	VAL173	PHE238	LEU294	LEU241
Compound	NSC217908	0	1	1	0	2	0	0	1	2
	NSC361563	0	1	2	0	2	0	0	2	2

Supplementary Figure 3. Pharmacological interactions of NSC217908 and NSC361563.
